# Journal of General Virology – Introduction to ‘ICTV Virus Taxonomy Profiles’

**DOI:** 10.1099/jgv.0.000686

**Published:** 2017-01-17

**Authors:** Andrew J Davison

**Affiliations:** MRC-University of Glasgow Centre for Virus Research, Sir Michael Stoker Building, 464 Bearsden Road, Glasgow G61 1QH, UK

**Keywords:** ICTV Virus Taxonomy Profiles

Virus classification is important because it provides a framework for understanding the amazing diversity of viruses. The authoritative resource in this area is the International Committee on Taxonomy of Viruses (ICTV) Report, which sets out the formal classification scheme in the context of extensive information on the viruses themselves.

The First ICTV Report was published in 1971, was 81 pages long and established 43 virus groups. Forty years later, the Ninth Report reached 1327 pages and described 87 families totalling 2284 species. The next Report will be even bigger but will no longer be produced as a book that has to be purchased – instead, it will be published online and made freely available to all. This will make it much easier for the ICTV to keep the information up to date by revising chapters or adding new ones as the taxonomy changes. It will also facilitate the inclusion of in-text references, clickable links and supplementary materials such as the sequence alignments used to produce the phylogenetic trees that are central to taxonomy.

In order to make sure that the new Report is accessible to literature search engines such as PubMed, the ICTV is pleased to have secured a partnership with the Microbiology Society to publish two-page summaries of the chapters in the Journal of General Virology under the heading ‘ICTV Virus Taxonomy Profiles’. The summaries will include brief descriptions of the structure, replication and taxonomy of members of each virus order and family, as well as permanent links leading to the full chapters at the ICTV website (http://ictv.global/report). These summaries will form the primary sources by which the Report chapters will be cited.

The new Report is being written by members of the ICTV Study Groups and edited by members of the ICTV Editorial Board. In addition, generous support from the Wellcome Trust through a Biomedical Resource Grant employs a Managing Editor and a Technical Editor to oversee the day-to-day process of chapter submission, review and publication. This grant also supported the first ever joint meeting of ICTV Study Group chairs and the ICTV Executive Committee (held at Hinxton, UK in February 2016), at which these new plans for the Report were presented and discussed. In addition, the grant has funded or will fund focused meetings on key issues in virus classification, including the role of metagenomics (held in Boston, USA in June 2016), the development of analytical methods and future strategies for virus classification.

Through the new publication format and the other planned developments, the ICTV will be able to respond more quickly to discoveries in virology, and the resources it produces will become available more readily to the scientific community and the public at large.

